# Perspectives of parents and adolescents on sexual and reproductive health information communication in Ghana

**DOI:** 10.4102/jphia.v16i1.845

**Published:** 2025-03-26

**Authors:** Frank B. Agyei, Doreen K. Kaura, Janet D. Bell

**Affiliations:** 1 Department of Nursing and Midwifery, Faculty of Medicine and Health Sciences, Stellenbosch University, Cape Town, South Africa

**Keywords:** parent, adolescent, sexual and reproductive health, information, communication

## Abstract

**Background:**

Parent-adolescent sexual and reproductive health (SRH) information communication is known to be associated with positive SRH outcomes of adolescents. This determinant of positive SRH outcomes of adolescents is therefore receiving attention in research in the African region.

**Aim:**

This study therefore explored and integrated the perspectives of parents and adolescents on SRH information communication in Ghana.

**Setting:**

The study was conducted at the Asante Akyem North Municipality of Ghana.

**Methods:**

A qualitative descriptive design was used to interview 10 parent-adolescent dyad. Participants were purposively recruited from the Asante Akyem North Municipality in Ghana. Thematic analysis was done inductively using Braun and Clarke’s approach. ATLAS.ti version 23.0.7 was used to store and manage data.

**Results:**

Three themes emerged, namely, SRH information communicated by parents and their adolescents, motivation for communicating SRH information, and SRH information communication skills. Some form of SRH information communication take place between parents and adolescents although they lack the requisite skills to communicate such information.

**Conclusion:**

An SRH information communication intervention, which is culturally appropriate, is needed to train both parents and adolescents to communicate SRH information in Ghana.

**Contribution:**

The study contributes to the understanding of SRH information communication by highlighting the influence of cultural norms, social support and parental engagement on adolescents’ access to accurate information, which can inform the development of culturally sensitive interventions to enhance SRH communication between parents and adolescents.

## Introduction

The World Health Organization (WHO) defines adolescents as individuals from age 10 years – 19 years, and these people constitute about 16% of the world’s population.^1^ In this period known as adolescence, young people experience physical, psychological and emotional changes that directly influence their sexual and reproductive health (SRH) and put them at risk of social problems as well.^2^ Globally, adolescents are increasingly becoming sexually active at an early age. In spite of advances in healthcare and education, adolescents are not well prepared to navigate issues of SRH at this stage. According to the United Nations Population Fund, almost 12 million female adolescents aged 15 years – 19 years give birth each year.^3^ Global incidence of sexually transmitted infections (STIs) among adolescents is rising with more than half of new cases occurring among this group of young people.^4^

Adolescents in lower- and middle-income countries face significant difficulties in addressing their SRH needs, with risky sexual behaviours being more common in these areas.^5^ They often experience challenges in obtaining SRH information from trustworthy sources, such as parents, who are generally their preferred source of guidance.

In sub-Saharan Africa, more than half of adolescents experience sexual encounters early.^6,7^ This early passage to sexual activities exposes them to risky sexual behaviours such as having multiple sexual partners, sexual coercion and sexual violence.^8^ Risky sexual behaviours among adolescents are associated with consequences such as STIs including human immunodeficiency viruses (HIV) and/or acquired immunodeficiency syndrome (AIDS), early and unwanted pregnancies, maternal deaths and unsafe abortion.^9^

In Ghana, adolescent pregnancies keep increasing.^10^ This may be attributed to the lack of SRH education, parental neglect and sexual abuse, among others.^11^ Furthermore, Ghana is known to have one of the highest rates of fertility among adolescents in West Africa, with the birth rates of adolescents standing at 75 births per 1000 women aged 15 years – 19 years.^3^ This highlights the urgent need to address adolescent SRH issues in the country.

Research from around the world has consistently demonstrated a link between adolescents’ understanding of SRH and their health outcomes. Insufficient access to accurate information is identified as a key factor that leads to risky sexual behaviours.^12^

In reducing the negative SRH outcomes among adolescents, one of the effective means is through sexuality education.^13,14^ It is known that open and informed SRH communication between parents and adolescents can delay sexual initiation, reduce risky sexual behaviours, avoid unwanted pregnancies, reduce STIs and contribute to the overall well-being of adolescents.^15^ Parents play a vital role in shaping the understanding of adolescents on SRH matters because they are the primary agents of socialisation, more especially in the Ghanaian context.^16^ However, in many Ghanaian families, there is limited SRH information communication between parents and adolescents because of cultural norms and taboos, which sometimes lead to misinformation as adolescents tend to get the information from unreliable sources.^17^

Some adolescents express the desire for information on SRH from their parents, but this desire does not meet the desire of parents to communicate with their adolescents on such issues. The disconnect between adolescents’ desire for information from their parents and the reluctance of parents in providing the information creates a gap that has remarkable implications for adolescent SRH outcomes.^2^

Encouraging parent-child communication as part of sexuality education is an effective way to drive lasting behavioural changes, particularly in reducing unintended pregnancies. Research shows that when parents are involved in educating their children about sexuality, it leads to reductions in STIs, early sexual activity and multiple sexual partners.^13,18^ Despite these benefits, many parents struggle to engage in conversations about sexuality because of feelings of embarrassment and discomfort.^19^

Parents are instrumental in teaching values, morals and standards that greatly influence adolescent sexual behaviour and decision-making, positioning them as key figures in shaping these behaviours.^20^ Communication between parents and adolescents on topics like relationships and sexual health has become a significant public health concern, as research shows it plays a vital role in establishing a strong foundation for healthy sexual behaviour and outcomes in adolescents.^2^

Given the importance of effective SRH communication in shaping adolescent behaviour and reducing risky sexual practices, it is essential to understand the factors that influence parent-adolescent SRH communication in Ghana. This study seeks to explore the perspectives of both parents and adolescents on SRH communication in Ghana. By integrating the views of both parents and adolescents, this research will provide valuable insights into how to foster more open and effective discussions about SRH, ultimately contributing to better health outcomes for adolescents in Ghana.

### Theoretical framework

**FIGURE 1:**
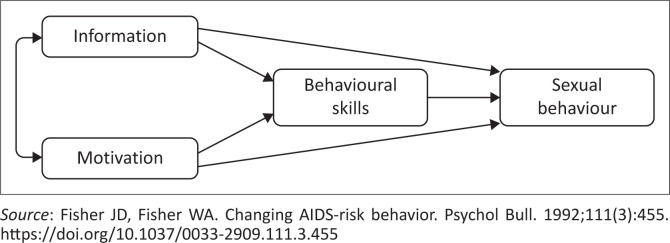
Original information, motivation and behavioural skills model.

The study is grounded in the Information, Motivation, and Behavioural Skills (IMB) Model (see Figure 1), which posits that effective health behaviours are driven by three key components: information, motivation and behavioural skills.^21^ The premise of the IMB model is that individuals who possess relevant health information, are motivated and have the necessary skills are more likely to engage in health-promoting behaviours, ultimately leading to positive health outcomes. Conversely, a lack of any of these elements may hinder the initiation and maintenance of such behaviours.

## Research methods and design

### Research design

This study used a qualitative descriptive design built on the principles of naturalistic inquiry and applicable in studies that are descriptive.^22,23^ The design allowed information to be gathered directly from those communicating SRH information and produced a comprehensive summary of it. The design was also considered because it makes it possible for the analysis to remain true to the voice of participants and enriches the transparency of the researcher’s interpretations.

### Study setting

The research was conducted in the Asante Akyem North Municipality of Ghana, comprising urban and rural settings. This area has seen a rise in migrants from across the country’s 15 regions, as well as some international migrants, creating a rich blend of ethnic and cultural diversity. Although the Akan culture, particularly the Asante customs, dominates, migrants from other regions maintain their cultural practices, often adapting to the prevailing Akan traditions. Many migrants also learn the local ‘Asante Twi’ language to facilitate communication. The main languages spoken in the municipality are English and ‘Asante Twi’, and it is home to 16 038 adolescents.^24^

### Participants and sampling strategy

In this study, parent-adolescent dyads were recruited, but interviewed separately to avoid parental influences on adolescents’ responses. Parents included biological fathers, mothers or male and female guardians of adolescents aged 13 years – 16 years who were willing to participate. The adolescents were selected using a purposive sampling approach, which targeted individuals in this age range. Flyers containing study information were distributed to potential participants, and informed consent was obtained from those who were willing to participate in the study. Parents signed consent forms, while adolescents provided assent prior to their inclusion.

To account for the developmental differences among adolescents, the authors purposefully included five parents of adolescents aged 13 years – 14 years and five parents of older adolescents aged 15 years – 16 years. The sampling also ensured maximum variability by including at least three male and three female parents. Adolescents who were cohabiting or married, along with their parents, were excluded from the study. Ultimately, data saturation was reached with a final sample of 10 parents and 10 adolescents.

### Data collection

The researcher and his assistants conducted individual interviews using a semi-structured interview guide. This guide was developed by the researcher in collaboration with the supervisory team, drawing on findings from a systematic review^25^ that was conducted in phase one of the study and the IMB skills model, with the aim of collecting data from both parents and adolescents. It included open-ended questions, along with prompts and probes, to encourage detailed and meaningful responses.

To test the guide’s clarity and the interview duration, a pilot interview was first conducted separately with a parent and their adolescent. The data from this pilot was transcribed, analysed and then reviewed with the supervisors, leading to minor adjustments to the guide to better explore the skills needed for SRH information communication.

Informed consent was obtained from the parents and child assent from the adolescents. Interviews were scheduled at locations and times that suited the participants. As most parents preferred interviews at home, both parent and adolescent interviews were conducted in the same setting, although separately, to avoid any influence of parental presence on the adolescents’ responses. The interviews were conducted face to face by the researcher and assistants, all of whom were experienced in qualitative interviewing. Consent was obtained to audio-record the conversations.

Each interview session lasted between 35 min and 60 min, involving only the participant, the researcher and the research assistants. Data were collected between August 2022 and January 2023.

### Data analysis

The data were analysed inductively using Braun and Clarke’s thematic analysis approach.^26^ The researcher first listened to the recorded interviews multiple times, both before and after transcription, to familiarise himself with the data. Interviews were transcribed verbatim in either ‘Asante Twi’ or English languages. For interviews conducted in Asante Twi, the transcripts were translated into English and then retranslated back into ‘Asante Twi’ language to ensure the participants’ perspectives were accurately captured. The researcher then cross-checked the transcripts against the audio recordings to verify transcription accuracy. Once verified, the transcribed data were uploaded into ATLAS.ti (version 23.0.7) for further organisation into meaningful units, which helped in generating initial codes. These codes were grouped to create themes and subthemes, which were refined to prevent any overlap. Conclusions were then drawn from the categories and themes, ensuring alignment with the study’s aim. Finally, the themes were defined, named and a report was produced based on the findings.

#### Trustworthiness

Throughout the processes, trustworthiness was ensured to accurately represent participants’ shared experiences. Credibility, confirmability, transferability and dependability formed the foundation for establishing trustworthiness in this study.^27^ Strategies such as utilising probes and prompts, conducting follow-up interviews and engaging in member checking were employed.

### Ethical considerations

Ethical clearance to conduct this study was obtained from the Health Research Ethics Committee (HREC) of the Stellenbosch University (S21/08/159) on 06 January 2022, and renewal and extension was further granted on 06 January 2023. Following this approval, the Committee on Human Research, Publication and Ethics of the College of Health Sciences of the Kwame Nkrumah University of Science and Technology granted approval in Ghana (CHRPE/AP/356/22) on 19 July 2023. This study adhered to the ethical guidelines set forth in the Declaration of Helsinki, which provides standards for research involving human participants.^28^

## Results

### Participants’ characteristics

Ten parent-adolescent dyads participated in the study. Among the 10 parents, four were males and six were females. Four out of the 10 adolescents were males and six were females. Participants were recruited from the Agogo (*n* = 5), Hwidiem (*n* = 3), Akutuase (*n* = 1) and Juansa (*n* = 1) communities in the municipality. Other participant characteristics are summarised in Table 1.

**TABLE 1:** Participants’ characteristics.

Parent (P)	Adolescent (A)	Age		Gender		Community	Tribe	Religion
		P	A	P	A			
P1	A5	38	15	F	F	Akutuase	Ashanti	Christianity
P2	A6	42	16	F	M	Agogo	Frafra	Islamic
P3	A4	76	14	F	F	Agogo	Ashanti	Christianity
P4	A8	42	14	M	F	Juansa	Akuapem	Christianity
P5	A9	39	13	F	F	Hwidiem	Ashanti	Christianity
P6	A7	36	13	F	M	Agogo	Ashanti	Christianity
P7	A10	40	15	M	M	Hwidiem	Fanti	Christianity
P8	A2	48	13	M	F	Hwidiem	Ashanti	Christianity
P9	A1	63	16	M	M	Agogo	Anlo Ewe	Christianity
P10	A3	50	16	F	F	Agogo	Dagomba	Islamic

### Themes generated from the study

Through exploring parent-adolescent SRH information communication in Ghana, three themes emerged with corresponding subthemes as summarised in Table 2 and further described. The three themes are SRH information communicated by parents and their adolescents, motivation to communicate SRH information, and SRH information communication skills.

**TABLE 2:** Themes and subthemes.

Themes	Subthemes
1. SRH information communicated by parents and adolescents	1.1. SRH topics communicated 1.2. Source of SRH information
2. Motivation to communicate SRH information	2.1. Parent and adolescent factors 2.2. Social factors
3. SRH information communication skills	3.1. Ability to communicate SRH information 3.2. Perceived self-efficacy

SRH, sexual and reproductive health.

#### Theme 1: Sexual and reproductive health information communicated by parents and adolescents

Sexual and reproductive health information communicated by parents and adolescents is the content of SRH that emerged to have been discussed by parents and their adolescents. It emerged that parents and adolescents do share information on SRH during their conversations. Two subthemes emerged here as SRH topics discussed and the source of information. This has been discussed below.

**Subtheme 1.1: Sexual and reproductive health topics communicated by parents and their adolescents:** It emerged that parents and their adolescents had shared information on changes during adolescence, menstruation, sex, abstinence, pregnancy and its consequences, STIs, abortion and virginity. These topics were not all discussed by each parent-adolescent dyad, but most were discussed by some parents and their adolescents.

A parent shared that the focus of his communication with his adolescent has been on changes during adolescence, hygiene during menstruation and abstinence:

‘Okay, the focus was on the changes in the adolescent, once she is entering her puberty age, with all the changes, the hormonal changes, the physical changes and attitudinal changes, we had a chat about them for her to be aware, the changes that are happening that is they are normal and she’s not ready for sex and other marital issues. The need for her to keep herself hygienically during menstruation.’ (P8, M, 48 years)

The daughter of this parent mentioned that:

‘Mmmm…the advice they give me to abstain myself and to avoid involving myself in any relationship till I am fully grown.’ (A2, F, 13 years)

Although she might have not remembered all of what her parents had been saying, she could mention abstinence and relationships, which was also captured in the parent’s response. This may indicate how pronounced the topic of abstinence is in the communication between the adolescent and her parents.

Others have talked about sex and pregnancy, but place emphasis on abstinence. One parent and her adolescent had this to say:

‘Eerrmm … sex, so far what we’ve told her is that if she engages in sex, she will get pregnant. But as for other things we have not talk that much, like STIs, and other … eerr … more of the times I relate the sex to pregnancy. I don’t know. But I think I want the abstinence, the abstinence. I want her to abstain from sex. That is why I don’t talk much about using of the contraceptives.’ (P2, F, 42 years)

The son mentioned that they have not discussed about contraceptives. He shared that:

‘My mother has not talked to me on contraceptives.’ (A6, M, 16 years)

The parent focuses primarily on linking sex to pregnancy and promotes abstinence, avoiding discussions about contraception, STIs and other topics. The adolescent confirms that contraceptives have not been discussed. This indicates a gap in communication, where other SRH issues are overlooked, potentially leaving the adolescent without comprehensive information.

**Subtheme 1.2: Source of sexual and reproductive health information:** This subtheme speaks to where parents and adolescents obtained the SRH information that they communicated. Information sources included Internet, television, radio, books for both parents and adolescents. Parents also used personal experiences, and adolescents also learned from their teachers in schools and parents at home. For example, a parent shared that:

‘I think it is from experience. As an adult or a married person, erhernn … So, it’s from experience. What I’ve learnt and also what I’ve also gather from other people especially in relationship and in my area or society. Sometimes I go to the internet, reading from what people have written about sexual reproductive health and also what I hear.’ (P4, M, 42 years)

This implies that the individual’s knowledge of SRH is shaped by both personal and social experiences, as well as information gathered from external sources like the Internet. This indicates a blend of informal learning from community interactions and self-directed education through modern platforms.

An adolescent participant also shared that:

‘TV, radio, friends, teachers and members in the community, I also hear that from my parents.’ (A8, F, 14 years)

The responses from the adolescent and the parent reflect the different sources of SRH information they rely on. The adolescent mentions receiving information from a wide variety of sources, including media (TV, radio), friends, teachers, community members and parents, indicating a broad network of influences. In contrast, the parent emphasises personal experience, knowledge from relationships, societal observations and even online resources as key sources of SRH information.

#### Theme 2: Motivation to communicate sexual and reproductive health information

These are the elements that influenced SRH information communication between parents and adolescents. Two themes emerged here as parent and adolescent factors and social factors (Table 2).

**Subtheme 2.1: Parent and adolescent factors:** These are the attitudes of parents and adolescents that had influence on their communication on SRH issues. Parents who viewed SRH information communication as important communicated with their adolescents and vice versa.

Some parents expressed that providing accurate information to adolescents is crucial, as withholding it may lead them to seek incorrect or misleading information from their peers. One shared that:

‘Because I believe, appropriateness of sexual reproductive health is based on the right information. Girls in their reproductive health, they are more close to your friends. So, if you take an information from a friend and it is a wrong one … so in order not to be influence by friends, I think it is important as parents, you need to do it and give the right information. Some people the information will make them bad … but for me it is the reverse, the more you know the more power you have to be able to help yourself.’ (P8, M, 48 years)

The adolescent also emphasised a preference for receiving accurate information from parents, expressing greater confidence in them over friends as a reliable source:

‘I share it with them because they can give me appropriate advice than friends or teachers. With what I have to do, I have to tell them so that they can advise me.’ (A2, F, 13 years)

Both the parent and adolescent emphasise the importance of receiving accurate SRH information from trusted sources. The parent believes it is essential to provide the right information to prevent adolescents from being misled by friends, viewing knowledge as empowering. Similarly, the adolescent values parental advice over that from friends or teachers, recognising their parents as a reliable source for guidance.

Some parents believe that exposing adolescents to information on SRH may lead them to engage in inappropriate behaviour, as they fear it could encourage experimentation. A parent said:

‘I felt like it is too much for her to get that lot of information because sometimes if you have such information, you intend to practice with them so I was afraid to go there.’ (P7, M, 40 years)

On the contrary, the adolescent of the same parent felt that receiving information on SRH is important for his well-being and informed decision-making. He believes that having access to SRH information will not encourage him to engage in such activities, but rather equip him to avoid them and make safer choices:

‘Please it is good because I will learn something, I will learn wisdom from it, so that I will move away from such things.’ (A10, M, 15 years)

**Subtheme 2.2: Social factors:** Social motivation relates to the influence of perceived social support and norms on parent-adolescent SRH information communication. Social motivation influences how parents and adolescents communicate about SRH. The level of perceived social support and prevailing norms either encouraged or discouraged these discussions. Parents and adolescents who felt supported by their social networks engaged in discussions about SRH.

Some participants mentioned that in their culture, there is a tendency to avoid discussing SRH matters with adolescents highlighting the influence of cultural norms on SRH communication practices. This was particularly evident among those parents who had not received formal education. A parent and his adolescent shared that:

‘In our culture, people as much as possible avoid especially the people who have not had this western education, if not, in my ethnic background you wouldn’t see parents doing that.’ (P9, M, 63 years)

‘Others do not openly talk about it here, if you talk it will look like you are the bad one and you know such things.’ (A1, M, 16 years)

Together, these statements highlight the cultural barriers to open communication, particularly around sensitive topics. They suggest that in certain communities, there is a reluctance to discuss such matters openly. This avoidance is rooted in traditional norms and the fear of social judgement, where discussing these topics may lead to being perceived negatively. Consequently, cultural and educational factors significantly shape how individuals engage in or avoid conversations, especially regarding sensitive issues.

Parents and adolescents highlighted that social support from significant others within the community is divided when it comes to discussing SRH with adolescents. Some parents and adolescents, lacking encouragement from other family members, avoid these conversations entirely. Meanwhile, others receive social support to engage in them. This reflects differing levels of social support influencing how parents approach important conversations with their children. A parent and her adolescent shared that:

‘Some people see it not to be good to talk about such things with adolescents. They don’t even think about it that when I advise my child it will be good, some don’t know and they leave their child to walk around anyhow. Others also see it to be good and they support that we discuss that.’ (P3, F,76 years)

‘Some say it’s good to talk about it. Other do not support such discussions. It depends on the person.’ (A4, F, 14 years)

Social support for discussing SRH topics varies based on individual perspectives and cultural norms. While some people view these discussions as beneficial, others may resist them because of societal or personal beliefs, making support for such conversations highly dependent on the person and their community.

#### Theme 3: Sexual and reproductive health information communication skills

One of the themes that emerged in the study is SRH information communication skills. This is the ability to effectively discuss and convey knowledge related to SRH topics. Two subthemes emerged: ability to communicate SRH information and perceived self-efficacy.

**Subtheme 3.1: Ability to communicate sexual and reproductive health information:** Parents and adolescents shared their experiences regarding their ability to communicate SRH information.

Some participants described a relaxed and open communication style with their adolescent, emphasising that their discussions feel natural and free-flowing rather than formal or rigid. They avoid strict or structured conversations, instead fostering a friendly and casual atmosphere where communication happens as part of their regular interactions. This approach helps maintain a comfortable, easy-going dialogue, making it easier to talk about sensitive topics without tension or discomfort:

‘We talk, we laugh, so it is like normal conversation. It is not something strict, I don’t sit her down like strict, no no no no, we just communicate as we have been doing always.’ (P5, F, 39 years)

‘We sat comfortably to discuss this. We also talked about teenage pregnancy. As for that one I was on my bed reading my book, we kept talking about it and I fell asleep.’ (A9, F, 13 years)

Some parents feel confident and at ease when communicating with the adolescent, often using humour to initiate conversations, which helps create a relaxed environment. Some adolescents recognise and appreciate the parent’s easy-going approach, which allows them to engage in discussions as well:

‘I have already psyched myself how I’m going to do it as at times it is introduced in a form of joke so it comes to me naturally. I don’t have apprehension.’ (P9, M, 63 years)

However, despite the comfortable atmosphere, some adolescents still experience occasional fear or nervousness during these conversations, reflecting a difference in emotional responses between the parent and adolescent:

‘I’m able to communicate well just that I get scared.’ (A1, M, 16 years)

The parent feels comfortable discussing SRH with their adolescent, often introducing the topic in a casual, natural manner. However, the adolescent, while able to communicate, experiences fear or discomfort during these conversations. This suggests a disconnect, where the parent’s ease does not fully alleviate the adolescent’s anxiety. It highlights the importance of not only the parent’s comfort but also addressing the adolescent’s emotional concerns to create a safe, open communication environment.

**Subtheme 3.2: Perceived self-efficacy:** Some parents expressed challenges in discussing these topics with their adolescents, while others found it easier and faced no difficulties. Similarly, some adolescents struggled to communicate with their parents about SRH matters, whereas others were able to engage in these conversations more comfortably.

A parent and her adolescent narrated how easier it is for them to communicate on SRH matters. They noted that open communication is easier when there is a close, trusting relationship. They feel comfortable talking to each other, because they have built a bond, making SRH discussions straightforward. The parents indicated that communication was made easy with the adolescent because the adolescent felt they were free to speak with the parent because of their close relationship:

‘Nothing makes it difficult for me to talk with her (giggles) … I took her in when she was very small, and we are close, so it is less difficult, and she listens to my advice … so it easy because I’m free with her.’ (P3, F, 76 years)

The adolescent also emphasised the openness she experiences when she speaks to the mother. The adolescent feels that she is understood by the mother. She will easily talk to her and the mother is open to explain anything the adolescent needs to know about:

‘It is not difficult because if you are close with your parents, it is possible to tell them because they are your parent so … my mum like this she is easy to understand me or my sister so if anything, we just talk to her and she explain everything to us, everything we need to know.’ (A4, F, 14 years)

The responses from both the parent and the adolescent highlight a strong, open relationship between them, characterised by mutual trust and closeness. The parent feels at ease discussing topics, including SRH, due to the bond they share, and the adolescent echoes this sentiment, acknowledging that their closeness makes communication easier. Both emphasise the importance of understanding and openness, with the adolescent noting that the mother is approachable and willing to explain things when needed.

There was a situation where a parent felt comfortable communicating with her adolescent on SRH matters while the adolescent expressed feelings of unease and anxiety because of a lack of prior conversations about sensitive topics. The parents emphasised that effective communication with adolescents requires being approachable and open to discussing any topic with them:

‘So, when you chat with them, nicely, if anything they will ask, and you answer them … so, it easy because I’m free with them.’ (P2, F, 42 years)

Conversely, the adolescent described emotions attributed to the conversations where she felt uneasy and anxious the first time, which was difficult for him:

‘I felt uneasy, I felt anxious … this is because I have not been talked to about such matters before and it was difficult for me.’ (A6, M, 16 years)

This situation reflects a disconnect in communication between the parent and adolescent. While the parent perceives the conversation as easy, the adolescent feels challenged when discussing SRH issues with their parents. This discrepancy could stem from differences in comfort levels, expectations or understanding of the communication dynamics. It highlights the importance of fostering an open dialogue where both parties feel heard and understood, as well as the need for parents to create an environment that encourages their children to share their feelings and concerns.

## Discussion

This study examined and integrated the perspectives of both parents and adolescents regarding SRH information communication in Ghana. The findings reveal that SRH communication does take place between parents and their adolescents, with SRH knowledge and motivation playing a key role in shaping communication skills. However, while some parents perceived these discussions as occurring in an open and comfortable environment, their adolescents often expressed feeling uneasy or uncomfortable during these interactions. This disconnect underscores the complexities in parent-adolescent communication, highlighting the need for more effective strategies to bridge these gaps.

### Information communicated

It emerged that parents and their adolescents had shared information on changes during adolescence, menstruation, sex, abstinence, pregnancy and its consequences, STIs, abortion and virginity. This is consistent with research both in Ghana and globally. In Ghana, studies indicate that parents frequently discuss issues like menstruation and abstinence, particularly with daughters, because of cultural beliefs about the importance of purity and the consequences of early sexual activity.^29,30,31^ This aligns with broader cultural norms that emphasise sexual morality and the dangers of premarital sex. Similar patterns of communication have been observed in studies in other parts of the world. Studies conducted in the United States^32^ and in India^33^ highlight that parents often engage in conversations about menstruation, puberty and abstinence, particularly with girls, because of societal expectations around female sexual behaviours. These discussions are aimed at preventing teenage pregnancy and STIs, similar to the concerns expressed by Ghanaian parents. In Western Pennsylvania, parents also prioritise conversations about abstinence and sexual consequences, often informed by the high rates of adolescent pregnancies and HIV.^34^ This highlights the need for comprehensive SRH education that facilitates open conversations about all aspects of sexual health, enabling adolescents to make informed decisions.

Information sources included Internet, television, radio, books for both parents and adolescents. Parents also used personal experiences, and adolescents also learned from their teachers in schools and parents at home. The implication is that although both parties are accessing SRH information, their sources and approaches may differ, possibly contributing to misunderstandings or discomfort in parent-adolescent communication about SRH. It highlights the need for parents to adapt their communication methods to be more aligned with the diverse sources that adolescents are exposed to. Similar trends have been reported in other studies in Ghana.^35,36^ This trend is echoed in Kenya, where it was noted that adolescents seek information from multiple sources, including school-based health programmes, but parents often struggle to provide accurate information because of cultural taboos.^37^ Similar patterns are seen in Egypt^38^ where it was reported that adolescents often seek sexual health information online, although concerns about misinformation exist.^39^ Overall, these findings highlight the need for targeted educational interventions that empower parents to communicate effectively about SRH topics while ensuring adolescents have access to reliable information. Encouraging open family dialogues can bridge knowledge gaps and promote healthier decision-making among young people.

### Motivation for sexual and reproductive health information communication

It was found that the perception of SRH information communication is important and significantly influences the interactions between parents and adolescents. Many parents recognise that providing accurate information is essential to prevent adolescents from seeking unreliable or misleading information from peers. This aligns with other studies in Ghana,^35^ which highlight that adolescents often turn to friends for SRH information when parents are not communicative, leading to potential misinformation.

Adolescents, in turn, express a preference for obtaining accurate information from their parents, viewing them as more reliable sources than peers. This sentiment mirrors a study, which found that adolescents are more likely to trust and seek out parental guidance when parents communicate openly about SRH topics.^40^ This trust fosters a healthier dialogue, enabling adolescents to make informed decisions regarding their sexual health.

However, some parents harbour concerns that discussing SRH may inadvertently encourage risky behaviours. This fear is supported by a study in Uganda where parents expressed apprehensions that open discussions about sexual health might promote promiscuity among adolescents.^41^ While the findings from Ghana affirm the importance of parental communication and the adolescents’ preference for accurate information, they also highlight a common parental fear regarding the potential negative consequences of discussing SRH topics. This reflects broader patterns observed in studies from other contexts, indicating a need for interventions that address these fears while promoting open, informative discussions about SRH.

Moreover, the influence of social support and cultural norms on SRH communication between parents and adolescents is a key factor in determining whether these conversations take place. In this study, parents and adolescents noted that social support within the community is often divided, with some parents and adolescents receiving encouragement to discuss SRH topics while others, lacking such support, avoid these conversations altogether. This divide reflects a broader cultural context in which social norms play a crucial role in shaping communication behaviours. The findings in this study are consistent with existing literature, both in Ghana^42^ and other parts of Africa.^43^ Meanwhile, in European countries such as the Netherlands, parents tend to feel more supported and culturally encouraged to discuss a broader range of SRH topics with adolescents, reflecting a more open dialogue.^44^ While some communities encourage open discussions, others maintain traditional views that create barriers to communication. This highlights the need for culturally sensitive interventions that address the social and cultural factors that inhibit SRH discussions.

### Sexual and reproductive health information communication skills

When it comes to SRH information communication skills, parents in this study used a relaxed, humorous approach to talk about SRH with their adolescents, which made them feel comfortable. However, some adolescents still experienced nervousness during these discussions. This finding is consistent across different countries. In the U.S.,^45^ parents’ casual communication styles helped open dialogue, but adolescents still felt uncomfortable discussing sensitive topics. In contrast to this, it was found that Dutch adolescents were at ease talking about SRH matters with their parents because of the culture of open communication around sexuality.^44^ This contrasts with the Ghanaian findings, where adolescents, despite their parents’ casual approach, still experience apprehension, which is potentially because of stronger cultural taboos and the sensitive nature of SRH discussions within more conservative social frameworks. These discrepancies highlight how cultural norms surrounding SRH communication can differ, with more progressive and open societies promoting greater ease in such conversations compared to more conservative or traditional settings like Ghana.

Also in the current study, parents found it challenging to discuss SRH topics with their adolescents, while others felt comfortable and faced no difficulties. Similarly, some adolescents struggled to communicate about SRH matters, whereas others engaged in these conversations more easily. This pattern mirrors findings in Ghana, where many parents hesitate due to discomfort.^42^ Internationally, similar challenges were noted in China, where cultural taboos often hinder discussions,^46^ and in Australia, where some parents felt equipped to talk about SRH while others struggled.^47^ These findings suggest a common trend across cultures, indicating the need for interventions to support effective SRH communication between parents and adolescents.

### Strengths and limitations

The study’s strength lies in its inclusion of diverse ethnic groups, which enhances the understanding of how cultural factors shape communication processes. The setting’s mix of migrants from urban and rural areas ensures that the findings are not overly representative of one particular environment. The researcher took care to minimise the impact of personal biases related to cultural and professional affiliations on data interpretation, actively seeking reflections and supervisory input throughout the process to ensure a balanced analysis. The interview guide was developed based on the findings from a systematic review of effective interventions conducted in the first phase of the study. Additionally, it was informed by the IMB model to ensure a comprehensive approach to data collection.

While the findings offer valuable theoretical and practical insights for developing a culturally tailored SRH information communication intervention, it is important to acknowledge certain limitations that should be considered when interpreting the results.

The study focused exclusively on adolescents aged 13 years – 16 years, which means that the parents involved were those with children in this age range. Consequently, the communication experiences of these parents and adolescents may not be applicable to those with adolescents older than these, even though similar experiences might have been encountered when those adolescents were within the study’s age group.

However, the sample was selected because the participants had experienced early adolescence and were transitioning to older adolescence. Given that SRH is a delicate subject, concerns regarding privacy and potential embarrassment may have led some parents and adolescents to withhold their true feelings, impacting how they shared their experiences. Nonetheless, measures were taken to ensure confidentiality, and adolescents were encouraged to speak openly about their experiences.

## Conclusion

The study reveals the challenges of SRH information communication between parents and adolescents in Ghana. While parents discuss puberty and abstinence, they often avoid contraception because of cultural beliefs and fears of promoting risky behaviour. This highlights the influence of social support and cultural norms, with some parents using casual approaches to foster dialogue, yet adolescents still feel apprehensive.

Overall, the findings emphasise the need for culturally sensitive educational interventions to enhance SRH communication skills of parents and adolescents. By addressing fears and encouraging open dialogue, these initiatives can empower adolescents to make informed decisions about their SRH.

### Recommendations

To enhance communication about SRH between parents and adolescents, it is recommended to develop training programmes for parents and adolescents focused on effective communication strategies to address discomfort and fears. Promoting open dialogue within families by creating safe spaces for discussions is essential, along with implementing culturally sensitive initiatives that tackle local norms and beliefs. Encouraging adolescents to seek information from parents can help counter misinformation while engaging community leaders can shift cultural attitudes towards healthier communication.

## References

[1] World Health Organisation. Adolescent health [homepage on the Internet]. 2023 [cited 2024 Sep 23]. Available from: https://www.who.int/health-topics/adolescent-health#tab=tab_13

[2] Usonwu I, Ahmad R, Curtis-Tyler K. Parent–adolescent communication on adolescent sexual and reproductive health in sub-Saharan Africa: A qualitative review and thematic synthesis. Reprod Health. 2021;18(1):202. 10.1186/s12978-021-01246-0 34629082 PMC8504018

[3] United Nations Population Fund. Adolescents and youth dashboard [homepage on the Internet]. 2022 [cited 2024 Sep 07]. Available from: https://www.unfpa.org/data/dashboard/adolescent-youth

[4] World Health Organization. Global health sector strategy on sexually transmitted infections (STIs) 2016–2021 [homepage on the Internet]. Geneva: WHO; 2020 [cited 2024 Sep 07]. Available from: https://iris.who.int/bitstream/handle/10665/246296/WHO-RHR-16.09-eng.pdf?sequence=1&isAllowed=y

[5] Prata N, Weidert K. Adolescent sexual and reproductive health. In: McQueen D, editor. Oxford research encyclopedia of global public health. Oxford: Oxford University Press, 2020; p. 1–24.

[6] Dagnachew Adam N, Demissie GD, Gelagay AA. Parent-adolescent communication on sexual and reproductive health issues and associated factors among preparatory and secondary school students of Dabat Town, Northwest Ethiopia. J Environ Public Health. 2020;2020(1):4708091. 10.1155/2020/4708091 32774393 PMC7397411

[7] Tabong PT, Maya ET, Adda-Balinia T, et al. Acceptability and stakeholders perspectives on feasibility of using trained psychologists and health workers to deliver school-based sexual and reproductive health services to adolescents in urban Accra, Ghana. Reprod Health. 2018;15(1):122. 10.1186/s12978-018-0564-x 29976216 PMC6034281

[8] Odo AN, Samuel ES, Nwagu EN, Nnamani PO, Atama CS. Sexual and reproductive health services (SRHS) for adolescents in Enugu state, Nigeria: A mixed methods approach. BMC Health Serv Res. 2018;18(1):92. 10.1186/s12913-017-2779-x 29422062 PMC5806240

[9] Gautam A, Sharma K, Dhakal S, Dhakal S, Chand A. Adolescent-parent communication on sexual and reproductive health and its associated factors among higher secondary school students of Tokha Municipality, Nepal: A cross-sectional study. J Public Health Res Community Health Dev. 2023;7(1):11–20. 10.20473/jphrecode.v7i1.39509

[10] Quist E. Teenage pregnancy in Ghana. YEN.COM.GH; 2021 [cited 2024 Sep 07]. Available from: https://yen.com.gh/186213-teenage-pregnancies-recorded-2020-ghana-covid-19-cases-2020-2021-combined.html

[11] Tseganu S. Sexual exploitation during lockdown in Ghana [homepage on the Internet]. World Vision Ghana; 2020 [cited 2024 Sep 07]. Available from: https://www.wvi.org/stories/ghana/sexual-exploitation-during-lockdown-ghana

[12] Igras SM, Macieira M, Murphy E, Lundgren R. Investing in very young adolescents’ sexual and reproductive health. Glob Public Health. 2014;9(5):555–569. 10.1080/17441692.2014.908230 24824757 PMC4066908

[13] Pleaner M, Milford C, Kutywayo A, Naidoo N, Mullick S. Sexual and reproductive health and rights knowledge, perceptions, and experiences of adolescent learners from three South African townships: Qualitative findings from the Girls Achieve Power (GAP Year) trial. Gates Open Res. 2022;6:60. 10.12688/gatesopenres.13588.2 37249954 PMC10220247

[14] Ziaei T, Gorji MG, Behnampour N, Aval MR. Effect of group consultation based on maternal communication skills on the perspectives of 13–15-year-old girls about sex dialogues. Biosci Biotechnol Res Commun. 2017;10:433–440.

[15] Kumi-Kyereme A, Awusabo-Asare K, Biddlecom AE, Tanle A. Influence of social connectedness, communication and monitoring on adolescent sexual activity in Ghana. Afr J Reprod Health. 2014;18(3):16–24.20698062

[16] Agyei FB, Kaura DK, Bell JD. Exploring the culturally sensitive sexual and reproductive health information communication skill needs of parents in Ghana. Afr J Prim Health Care Fam Med. 2023;15(1):4101. 10.4102/phcfm.v15i1.4101 37916722 PMC10623484

[17] Reuben AA, Bagrmwin L, Ndanu TA, Aniteye P. Sexual and reproductive health communication between parents and adolescents: The case of Wa West District of the Upper West Region, Ghana. Health Sci Invest J. 2023;4(1):457–464. 10.4102/phcfm.v15i1.4101

[18] Bonjour M, Van der Vlugt I. Comprehensive sexuality education. Knowledge File. 2018;2:1–35.

[19] Rodgers KB, Tarimo P, McGuire JK, Diversi M. Motives, barriers, and ways of communicating in mother-daughter sexuality communication: A qualitative study of college women in Tanzania. Sex Educ. 2018;18(6):626–639. 10.1080/14681811.2018.1451988

[20] Sejati PE, Mufida RT, Wulandari A. Barrier to parents-adolescent communication on sexual and reproductive health to prevent premarital sexual behaviour: A qualitative systematic review. J Qual Womens Health. 2024;7(1):28–36. 10.30994/jqwh.v7i1.238

[21] Fisher JD, Fisher WA. Changing AIDS-risk behavior. Psychol Bull. 1992;111(3):455. 10.1037/0033-2909.111.3.455 1594721

[22] Kim H, Sefcik JS, Bradway C. Characteristics of qualitative descriptive studies: A systematic review. Res Nurs Health. 2017;40(1):23–42. 10.1002/nur.21768 27686751 PMC5225027

[23] Sandelowski M. Whatever happened to qualitative description? Res Nurs Health. 2000;23(4):334–340. 10.1002/1098-240X(200008)23:4<334::AID-NUR9>3.0.CO;2-G 10940958

[24] Ghana Statistical Service. 2010 Population & housing census report: Disability in Ghana. Accra: Ghana Statistical Service; 2014.

[25] Agyei FB, Kaura DK. A systematic review of effective parent-adolescent sexual and reproductive health information communication in lower-and middle-income countries. Health SA Gesondheid. 2023;28:2435. 10.4102/hsag.v28i0.2435 38076672 PMC10699210

[26] Braun V, Clarke V. What can ‘thematic analysis’ offer health and wellbeing researchers. Int J Qual Stud Health Well-Being. 2014;9(1):26152. 10.3402/qhw.v9.26152 25326092 PMC4201665

[27] Guba EG, Lincoln YS. Competing paradigms in qualitative research. Handbook Qual Res. 1994;2(163–194):105.

[28] World Medical Association. Declaration of Helsinki: Ethical principles for medical research involving human subjects. JAMA. 2013;310(20):2191–2194. 10.1001/jama.2013.281053 24141714

[29] Asampong E, Osafo J, Bingenheimer JB, Ahiadeke C. Adolescents and parents’ perceptions of best time for sex and sexual communications from two communities in the Eastern and Volta Regions of Ghana: Implications for HIV and AIDS education. BMC Int Health Hum Rights. 2013;13:40. 10.1186/1472-698X-13-40 24070548 PMC3849363

[30] Baku EA, Agbemafle I, Adanu RM. Effects of parents training on parents’ knowledge and attitudes about adolescent sexuality in Accra Metropolis, Ghana. Reprod health. 2017;14(1):101. 10.1186/s12978-017-0363-9 28836984 PMC5571628

[31] Nketia R. Parent-adolescent communication on sexual and reproductive health: Perceptions and experiences of out-of-school adolescent mothers in the East Gonja Municipality, Ghana. Int J Multidiscipl Stud Innov Res. 2022;10(3):1589–1601. 10.53075/Ijmsirq/746464353242

[32] Flores D, Barroso J. 21st century parent–child sex communication in the United States: A process review. J Sex Res. 2017;54(4–5):532–548. 10.1080/00224499.2016.1267693 28059568 PMC5808426

[33] Guilamo-Ramos V, Soletti AB, Burnette D, Sharma S, Leavitt S, McCarthy K. Parent–adolescent communication about sex in rural India: US–India collaboration to prevent adolescent HIV. Qual Health Res. 2012;22(6):788–800. 10.1177/1049732311431943 22232297 PMC3343220

[34] Ramchandani K, Morrison P, Gold MA, Akers AY. Messages about abstinence, delaying sexual debut and sexual decision-making in conversations between mothers and young adolescents. J Pediatr Adolesc Gynecol. 2018;31(2):107–115. 10.1016/j.jpag.2017.10.007 29097292 PMC5866200

[35] Challa S, Manu A, Morhe E, et al. Multiple levels of social influence on adolescent sexual and reproductive health decision-making and behaviors in Ghana. Women Health. 2018;58(4):434–450. 10.1080/03630242.2017.1306607 28296626 PMC5891210

[36] Adzovie DE, Adzovie RH. Exploring Ghanaian adolescent sexual and reproductive health (SRH) information source(s): A qualitative approach. Qualitative Report. 2022;27(3):648–663. 10.46743/2160-3715/2022.5126

[37] Macharia P, Pérez-Navarro A, Inwani I, Nduati R, Carrion C. An exploratory study of current sources of adolescent sexual and reproductive health information in Kenya and their limitations: Are mobile phone technologies the answer? Int J Sex Health. 2021;33(3):357–370. 10.1080/19317611.2021.1918311 38595745 PMC10929578

[38] Stevens R, Gilliard-Matthews S, Dunaev J, Todhunter-Reid A, Brawner B, Stewart J. Social media use and sexual risk reduction behavior among minority youth: Seeking safe sex information. Nurs Res. 2017;66(5):368–377. 10.1097/NNR.0000000000000237 28858145 PMC5661993

[39] Ibegbulam IJ, Akpom CC, Enem FN, Onyam DI. Use of the Internet as a source for reproductive health information seeking among adolescent girls in secondary schools in Enugu, Nigeria. Health Info Libr J. 2018;35(4):298–308. 10.1111/hir.12242 30426642

[40] Widman L, Choukas-Bradley S, Noar SM, Nesi J, Garrett K. Parent-adolescent sexual communication and adolescent safer sex behavior: A meta-analysis. JAMA Pediatrics. 2016;170(1):52–61. 10.1001/jamapediatrics.2015.2731 26524189 PMC4857605

[41] Ndugga P, Kwagala B, Wandera SO, Kisaakye P, Mbonye MK, Ngabirano F. ‘If your mother does not teach you, the world will…’: A qualitative study of parent-adolescent communication on sexual and reproductive health issues in border districts of eastern Uganda. BMC Public Health. 2023;23(1):678. 10.1186/s12889-023-15562-6 37041536 PMC10088803

[42] Baku EA, Agbemafle I, Kotoh AM, Adanu RM. Parents’ experiences and sexual topics discussed with adolescents in the Accra Metropolis, Ghana: A qualitative study. Adv Public Health. 2018;2018(1):5784902. 10.1155/2018/5784902

[43] Mbugua SM, Karonjo JM. Reproductive health knowledge among college students in Kenya. BMC Public Health. 2018;18:907. 10.1186/s12889-018-5760-7 30041605 PMC6057010

[44] De Looze M, Constantine NA, Jerman P, Vermeulen-Smit E, ter Bogt T. Parent–adolescent sexual communication and its association with adolescent sexual behaviors: A nationally representative analysis in the Netherlands. J Sex Res. 2015;52(3):257–268. 10.1080/00224499.2013.858307 24512029

[45] Wilson EK, Dalberth BT, Koo HP, Gard JC. Parents’ perspectives on talking to preteenage children about sex. Perspect Sex Reprod Health. 2010;42(1):56–63. 10.1363/4205610 20415887

[46] Wang N. Parent-adolescent communication about sexuality in Chinese families. J Fam Commun. 2016;16(3):229–246. 10.1080/15267431.2016.1170685

[47] Morawska A, Walsh A, Grabski M, Fletcher R. Parental confidence and preferences for communicating with their child about sexuality. Sex Educ. 2015;15(3):235–248. 10.1080/14681811.2014.996213

